# Reducing test request for anti-thyroglobulin and anti-thyroid peroxidase antibodies: trends before and after interventions based on rejection rules and profile management

**DOI:** 10.11613/BM.2018.030709

**Published:** 2018-10-15

**Authors:** Pedro Sánchez Pellicer, Ruth González Tamayo, Vicente Navarro López

**Affiliations:** 1 Unilabs Laboratory Clinical Biochemistry, Vinalopó University Hospital, Elche, Spain; 2 Department of Clinical Medicine, MiBioPath group, Universidad Católica San Antonio de Murcia (UCAM), Murcia, Spain; 3 Unilabs Laboratory Clinical Biochemistry, Torrevieja University Hospital, Torrevieja, Spain; 4 Unit of Infectious Diseases, Vinalopó University Hospital, Elche, Spain

**Keywords:** demand management laboratory, test laboratory request, anti-thyroid antibodies, anti-thyroid peroxidase antibodies, anti-thyroglobulin antibodies

## Abstract

**Introduction:**

The objective of this study was to identify trends in requests for anti-thyroid peroxidase antibodies (TPOab) and anti-thyroglobulin antibodies (TGab) tests before and after applying set of interventions based on rejection rules and profile management.

**Materials and methods:**

Trend analysis was made at semester time intervals (from May-October 2010 to May-October 2017), before and after the intervention semester (May-October 2016). Number of tests (N) TPOab and TGab / 1000 total requests based on total N of both tests and total N of biochemical analysis laboratory requests, was calculated. To find out where the interventions had more impact we distinguished N of requests between Primary Care (PC) and Specialized Care (SC). A joinpoint regression analysis was used to determine time segments and time points in these indicators where the trend changed.

**Results:**

Trend analysis of the request of TPOab and TGab showed two clearly differentiated trend lines with a statistically significant Joinpoint (P < 0.001) with an increase in each semester from May 2010 of 7.4% and 7.5% respectively, to the semester of the interventions where there was a decrease of - 45% and - 61% located mainly in PC. Trend analysis in SC setting did not show any Joinpoint and any trend line.

**Conclusions:**

Results showed that applied interventions enabled change of trend for TPOab and TGab test requests, especially in PC where the interventions proved to be the most successful.

## Introduction

Clinical decisions are mainly based on laboratory test results, and it is essential to keep demand for tests under control. Establishing strategies to ensure that the amount of requests for laboratory tests is appropriate should be a transversal measure proper to good management of any health centre (*1*).

A request for laboratory test could be considered “inappropriate” when it is not based on any scientific evidence. There are two possible situations: either the test is not justified by the given diagnosis or the clinical situation; and/or the time interval of a new request following a previous one is not suitable (*2*-*4*). This can result in additional and unnecessary costs. It is also recognized that test requesting varies significantly between different countries, regions and even among doctors belonging to the same hospital or service (*5*). An appropriate and timely requirement for a laboratory test improves quality and efficiency of healthcare. Therefore, it is necessary to carry out a trend analysis of requests over the years, verify if there is an increase in requests, analyse the causes, apply measures to adjust the numbers, and check the success of these interventions (*6*).

The determination of anti-thyroid antibodies, anti-thyroid peroxidase antibodies (TPOab) and anti-thyroglobulin antibodies (TGab), has widespread clinical use: autoimmune thyroid disease is among the most prevalent autoimmune diseases and the object of a large number of consultations both in Endocrinology and in the Primary Care (PC). The semiological values of these antibodies in thyroid autoimmune disease and in thyroid cancer have been included in clinical guidelines for some time, but demand for them varies greatly in routine medical practice (*7*). This can be due to multiple causes, for example opening the requests to non-endocrinologists such as internists and PC physicians (*8*). The objective of this study was to identify trends in requests for TPOab and TGab tests before and after applying set of interventions based on rejection rules and profile management.

## Materials and methods

### Study design

The study was conducted at the University Hospital of Vinalopó (Elche, Spain) from May 2010 to October 2017. This hospital has 200 beds and serves a population of about 150,000 inhabitants. The laboratory provides biochemical analysis and microbiology services. It is open 24 hours a day during which it ensures its complete range of services, which are not restricted to emergencies only.

The University Hospital of Vinalopó is equipped by information system and Electronic Health Records are used. The principle of test requesting in the hospital is that a search of particular laboratory tests by doctors could be made through hospital information system. Tests for TPOab and TGab could be requested separately as well as through the profiles named “Endocrine-Special Tests” and “Thyroid Cancer Study” respectively that was consisted of special non-routine tests for the study of endocrine pathology. All doctors from PC and Specialized Care (SC) have access and could request all laboratory tests from the catalogue through the hospital information system.

Requests for TPOab and TGab tests in period from May 2010 to October 2017 were collected from laboratory information system. In period from May 2010 to October 2017 number (N) of requests was observed through the semesters (periods from May-October and November-April) for each year of study. The period of May-October 2016 was defined as semester of interventions when we applied set of specific interventions.

1. In May 2016, an association of the TPOab and TGab tests, named anti-thyroid antibodies, was removed from the catalogue, so both had to be requested separately rather than simultaneously as before.
2. In October 2016, the profiles under which these tests appeared were restructured; TPOab test were included under the profile corresponding to special endocrinology tests, and TGab under the profile corresponding to study of differentiated thyroid cancer next to thyroglobulin. Furthermore, three rejection rules applied for test ordering:
  - TPOab: If a previous positive result has been found, the comment “POSITIVE result in previous analysis, determination is irrelevant since it is NOT useful in monitoring autoimmune thyroid disease” appears and the request is not processed (rejection rule 1).
  - TGab: Except in the case of thyroid cancer, if this test is requested together with the TPOab test, and the latter is positive, the following comment appears: “This test will not be performed since the determination of TGab does not provide any new information to detect autoimmune thyroid disease with respect to the POSITIVE TPOab” (rejection rule 2).
  - If both tests are requested together for the first time and the TPOab is positive, they are not performed either and the same comment appears (rejection rule 3). Thus, the test is only carried out if the TPOab test is negative. A separate comment is given in the case of diagnosis and monitoring of thyroid cancer, in which case the tests are performed in all cases.

The indicators that we use to monitor the demand and conduct a trend analysis study before and after these specific interventions in TPOab and TGab tests were the following:

N tests TPOab and TGab / 1000 total requests (TR) (TPOab / 1000 TR and TGab / 1000 TR) based on total N of both tests for numerator and total N of biochemical analysis laboratory requests for denominator.
N tests TPOab and TGab / 1000 PC requests (PCR) (TPOab / 1000 PCR and TGab / 1000 PCR) based on total N of both tests requested from PC for numerator and total N of biochemical analysis laboratory requests requested from PC for denominator.
N tests TPOab and TGab / 1000 SC requests (SCR) (TPOab / 1000 SCR and TGab / 1000 SCR) based on total N of both tests requested from SC for numerator and total N of biochemical analysis laboratory requests requested from SC for denominator.
Ratio TGab / TPOab total tests based on total N of both tests.

The indicator TPOab and TGab / 1000 TR will allow us to show the evolution of the global request of these tests and to verify if the measures were effective. TPOab and TGab / 1000 PCR and TPOab and TGab / 1000 SCR will allow us to assess where interventions have had more impact, SC or PC. Ratio TPOab / TGab total tests is an indicator of achieved objective to assess the success of interventions. If result is equal to 1 it means that tests are requested simultaneously.

### Statistical analysis

To determine trend of TPOab and TGab requests the specific indicators were used and joinpoint regression was performed for the each studied semester. Joinpoint regression is a statistical modelling technique that explains the relationship between two variables by means of a segmented linear regression constrained to be continuous everywhere, in particular in those places where the slope of the regression function changes. It is composed of a few continuous linear phases; line segments are joined at points called Joinpoints (*9*). Each Joinpoint shows a significant change in trend statistically significant (P < 0.05), and a Semester Percentage of Change (SPC) of indicators used in study with the corresponding 95% confidence interval (95% CI). The SPC were proposed to summarize and compare the rates of changes that are not constant over a given time period and shows the percentage (%) of change for each indicator in each trend line. A negative value (upper CI is negative) of SPC indicated a decreasing trend, whereas a positive value (lower CI is positive) of SPC indicated an increasing trend. A SPC was calculated by logarithmic transformation. It is usual assumption that the indicators were normally distributed, because the high numbers of numerators and denominators of indicators, and heteroscedastic variances for this. Montecarlo permutation test was used to prove the existence of Joinpoints. This permutation test is used for testing between two different joinpoint models, a simpler model with fewer Joinpoints called the null model, and a more complicated model called the alternative model (*9*).

Notably, the presence of one Joinpoint would imply two SPC, one before and one after, with two trend lines. The presence of any Joinpoint may or may not imply a single SPC: there will be one SPC if it is statistically significant from zero at the alpha level = 0.05.

In order to build the model, we used the free Joinpoint Regression Program Version 4.5.0.1 (National Cancer Institute, 2017).

## Results

**Figure 1:**
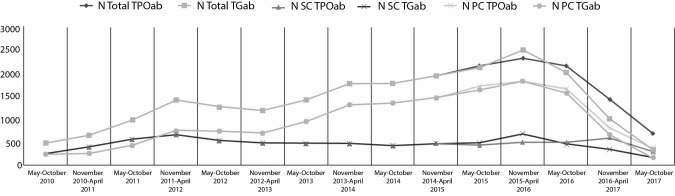
Number of antithyroid antibody test requests from May-October 2010 to May-October 2017. We represent in the vertical axis the number of anti-thyroid peroxidase antibodies (TPOab) and anti-thyroglobulin antibodies (TGab) tests from global, primary care and specialized care, and in the horizontal axis the semesters of study. N Total TPOab - total number of TPOab tests requested globally. N Total TGab - total number of TGab tests requested globally. N SC TPOab - number of TPOab tests requested from Specialized Care. N SC TGab - number of TGab tests requested from Specialized Care. N PC TPOab - number of TPOab tests requested from Primary Care. N PC TGab - number of TPOab tests requested from Primary Care.

In the semester previous to interventions, globally request for TPOab and TGab tests was 2347 and 2525 to decrease until 693 and 352, respectively, in last semester of study (Figure 1).

Global request for anti-thyroid antibodies tests, measured as TPOab / 1000 TR and TGab / 1000 TR, presented two clearly differentiated trend lines with a statistically significant Joinpoint (P < 0.001) in the semester of May to October 2016, that was semester of interventions. First segment presented an SPC of 7.4% (CI 95%: 9.8 to 5.1%) and 7.5% (CI 95%: 9.9 to 5.2%) for TPOab / 1000 TR and TGab / 1000 TR, respectively, and showing an increase in request of both tests since the opening of the hospital (May 2010). Second segment presented an SPC of - 45% (CI 95%: - 63 to - 19%) and - 61% (CI 95%: - 80 to 20%) respectively, and representing a decrease beginning with the start of interventions in May 2016 (Table 1). Notably, request was similar for both tests, almost exactly equal over the same period until semester of interventions; in fact, TGab / TPOab total tests was 1 until reaching Joinpoint revealing the change in trend. In last semester of study decreased until 0.4, a more appropriate number.

**Table 1:** Trend analysis of number of anti-thyroid peroxidase antibodies and anti-thyroglobulin antibodies tests *per* 1000 of total requests

**Semester of study**	**TPOab / 1000 TR**	**SPC of trend line TPOab / 1000 TR**	**TGab / 1000 TR**	**SPC of trend line TGab / 1000 TR**
May - October 2010	8.1	7.4% (CI 95%: 9.8 to 5.1%)	8.1	7.5% (CI 95%: 9.9 to 5.2%)
November 2010 - April 2011	7.4		7.4	
May - October 2011	10.6		10.5	
November 2011 - April 2012	14.0		14.0	
May - October 2012	12.5		12.5	
November 2012 - April 2013	10.8		10.8	
May - October 2013	12.6		12.6	
November 2013 - April 2014	15.0		15.0	
May - October 2014	15.5		15.5	
November 2014 - April 2015	17.0		17.0	
May - October 2015	19.4		19.1	
November 2015 - April 2016	20.0		21.5	
May - October 2016	18.8	- 45% (CI 95%: - 63 to - 19%)	17.6	- 61% (CI 95%: - 80 to 20%)
November 2016 - April 2017	12.1		8.6	
May - October 2017	6.0		3.0	
TPOab - anti-thyroid peroxidase antibodies. TGab - and anti-thyroglobulin antibodies. Results are presented as number of TPOab and TGab tests per 1000 of total requests through semesters from May 2010 to October 2017. Semester of interventions was May-October 2016. By joinpoint regression two lines of trend along with 2 SPS for both specific indicators were obtained. TR - total requests. SPC - semester percentage of change.				

In the semester previous to interventions PC request for both tests was 1840 to decrease until 396 TPOab tests and 161 TGab tests in last semester of study.

We find that in PC with TPOab / 1000 PCR and TGab / 1000 PCR indicators; also, there were two clearly differentiated trend lines with a statistically significant Joinpoint (P < 0.001) in both tests in the semester of interventions. First segment presented an SPC of 12.1% (CI 95%: 15.5 to 8.8%) for TPOab and 11.7% (CI 95%: 15.1 to 8.4%) for TGab, showing an increase in request since 2010. Second segment presented an SPC of - 58% (CI 95%: - 77 to - 24%) and - 70% (CI 95%: - 87 to - 29%) respectively, representing a decrease in PC in request for these tests (Table 2). Number of requested tests from PC decreased since semester of interventions as shown in Figure 1.

**Table 2:** Trend analysis of number of anti-thyroid peroxidase antibodies and anti-thyroglobulin antibodies tests *per* 1000 of primary care requests

**Semester of study**	**TPOab / 1000 PCR**	**SPC trend line TPOab / 1000 PCR**	**TGab / 1000 PCR**	**SPC trend line TGab / 1000 PCR**
May - October 2010	9.6	12.1% (CI 95%: 15.5 to 8.8%)	9.6	11.7% (CI 95%: 15.1 to 8.4%)
November 2010 - April 2011	7.8		7.8	
May - October 2011	12.4		12.4	
November 2011 - April 2012	21.0		21.0	
May - October 2012	20.5		20.5	
November 2012 - April 2013	19.1		19.1	
May - October 2013	24.3		24.3	
November 2013 - April 2014	32.4		32.4	
May - October 2014	33,0		33.0	
November 2014 - April 2015	35.9		35.9	
May - October 2015	41.2		39.3	
November 2015 - April 2016	41.8	- 58% (CI 95%: - 77 to - 24%)	41.8	- 70% (CI 95%: - 87 to 29%)
May - October 2016	39.2		37.0	
November 2016 - April 2017	18.4		15.0	
May - October 2017	9.3		3.8	
TPOab - anti-thyroid peroxidase antibodies. TGab - and anti-thyroglobulin antibodies. Results are presented as number of TPOab and TGab tests per 1000 of primary care requests through semesters from May 2010 to October 2017. Semester of interventions was May-October 2016. By joinpoint regression two lines of trend along with 2 SPS for both specific indicators were obtained. PCR - primary care requests. SPC - semester percentage of change.				

In SC setting Joinpoint was not found. Therefore, there was no trend line and any trend change in TPOab and TGab test request because both SPCs were not statistically significant from zero at the alpha level = 0.05. Despite intervention, N of requested tests from SC remain at the same level as shown in Figure 1.

Rejection rule designed for TPOab test produced a rejection rate of 16% since its implementation. The rest of the reduction was due to modifications to the profiles. In case of TGab tests our 2 rejection rules designed generated a 17% rejection rate since its implementation. Rest of the reduction was attributed to fact of categorizing the test under a specific profile for the study of differentiated thyroid cancer with thyroglobulin.

## Discussion

Results obtained showed a significant global decrease in both TPOab and TGab tests after applying set of interventions based on rejection rules and profile management that began in the semester of May to October 2016. The intervention applied, was successful in PC, whereas no change in trend was obtained in SC.

In the study of Salinas *et al.* obtained ratio of TGab / TPOab total tests median was 0.7, based on data from 110 Laboratories in Spain in 2014 (*8*). It was an observational study, and we do not know if in each of these laboratories any interventions were applied to manage request for TPOab and TGab tests. In our study, ratio TGab / TPOab total tests was quite below this observed median in the period post intervention. Thus, the department requests a relatively low number of tests, compared to other regions of Spain, and this was an indication that request in last semester of study was more appropriate.

Due to great variations in number of laboratory test requests found in previous studies in PC, we decided to differentiate the study of requests in PC and SC (*10*). Regarding PC, we observed a trend profile similar to that of global request trend, though more accentuated, with a higher SPC in pre- and post-intervention phases. This means that requests were increasing and then decreased significantly, implying that interventions put in place were clearly successful for both tests. Primary Care concentrated an inappropriate number of TPOab and TGab tests, in this case, and we improved this by provided interventions.

The study of requests for TPOab and TGab tests in SC did not show any Joinpoint statistically significant. The N of both tests has been relatively stable since 2010 and interventions had no effect on N of requests. Thus, it could be concluded that SC could be more familiar with the handling of these tests and aware on recommendations.

The main limitation of our study is that applied successful interventions not guarantee that it was safe. We thought that interventions were safe from point of view that they were more appropriate, but we do not have data about indicators that confirm this data, such as the number of new cases diagnosed of thyroid disorders or the increase of sequelae due to untreated/undiagnosed thyroid disorders (*11*). We thought that it could be the object of a new study.

There was a wide range of possible interventions to adapt test request and the literature shows that their impact can differ. It is difficult to pinpoint the most effective interventions. However, it is essential to dispose electronic laboratory requests and to perform multiple interventions simultaneously (*2*). In a recent systematic review, effectiveness of different possible interventions to reduce unnecessary requests for thyroid tests, including tests for TPOab and TGab antibodies was assessed (*12*). General conclusion was that interventions are effective when request process is modified, because clinical guidelines are better applied; but the review does not establish which interventions are the most effective because they were rarely reported in the studies. In this sense, our work attempted to make the interventions explicit, in order to make the study as reproducible as possible. We believe that methodology we described is a highlight of our study, as it contributes to the evaluation of request histories of laboratory tests and impact of performed interventions.

One year passes from the end of the semester of interventions in November 2016 until the end of the study in October 2017 and we thought that it was a considerable time to consider our conclusions as valid. Obviously, we expect the trend to continue over time as it adapts a jointpoint regression model. More specifically, recent semester data indicate that trend in request for these tests still continues to decrease (unpublished data).

Tests for TPOab would be recommended for the diagnosis and to detect a risk factor of autoimmune thyroid disease, as well as a risk factor for thyroid dysfunction in other situations such as pregnancy, certain medications, Down syndrome, *etc* (*7*). The aim of our interventions was to end practice of requests when following-up autoimmune thyroid disease, as previous positive tests exist. On the other hand, TGab tests are recommended in cases of differentiated thyroid cancer together with determination of thyroglobulin, as well as for diagnosing autoimmune thyroid disease in areas deficient in iodine (*7*). In areas with sufficient iodine, such as context of this study, it is not recommended to determine both antibodies since patients with negative TPOab tests and detectable TGab rarely have thyroid dysfunction (*13*). Interventions carried out on TGab requests were designed to prevent both antibodies from being requested simultaneously, by modifying the profiles; if both were requested, the objective was to cancel the TGab if the TPOab test was detected positive first. Due to all these interventions, a negative slope trend was achieved in the global demand for TPOab and TGab tests with an SPC of - 45% and - 61%, respectively. Thus, results showed that applied intervention enabled change of TPOab and TGab test requests overuse as well as adjusting appropriate requests for both test. A key setting where the interventions were successful was PC, but test request in SC was not modified. We recommend that interventions for the correct request for TPOab and TGab tests should be focused on PC.
